# Identification of four *TTN* variants in three families with fetal akinesia deformation sequence

**DOI:** 10.1186/s12920-024-01946-z

**Published:** 2024-06-27

**Authors:** Lihong Fan, Haibo Li, Ying Xu, Yingzhi Huang, Yeqing Qian, Pengzhen Jin, Xueping Shen, Zhi Li, Mingsong Liu, Yufei Liang, Guosong Shen, Minyue Dong

**Affiliations:** 1 Center of Prenatal Diagnosis, Huzhou Maternity & Child Health Care Hospital, No. 2 East Street, Wuxing district, Huzhou, 313000 Zhejiang China; 2 Central Laboratory of Birth Defects Prevention and Control, Ningbo Women and Children’s Hospital, Ningbo, China; 3 Women’s Hospital, School of Medicine, Zhejiang University, No.1 Xueshi road, Shangcheng district, Hangzhou, 310006 Zhejiang China; 4 Key Laboratory of Reproductive Genetics (Zhejiang University), Ministry of Education, Hangzhou, China

**Keywords:** *TTN*, Whole-exome sequencing, Fetal akinesia deformation sequence (FADS), RT-PCR, Meta transcript-only

## Abstract

**Background:**

*TTN* is a complex gene with large genomic size and highly repetitive structure. Pathogenic variants in *TTN* have been reported to cause a range of skeletal muscle and cardiac disorders. Homozygous or compound heterozygous mutations tend to cause a wide spectrum of phenotypes with congenital or childhood onset. The onset and severity of the features were considered to be correlated with the types and location of the *TTN* variants.

**Methods:**

Whole-exome sequencing was performed on three unrelated families presenting with fetal akinesia deformation sequence (FADS), mainly characterized by reduced fetal movements and limb contractures. Sanger sequencing was performed to confirm the variants. RT-PCR analysis was performed.

**Results:**

*TTN* c.38,876–2 A > C, a meta transcript-only variant, with a second pathogenic or likely pathogenic variant *in trans*, was observed in five affected fetuses from the three families. Sanger sequencing showed that all the fetal variants were inherited from the parents. RT-PCR analysis showed two kinds of abnormal splicing, including intron 199 extension and skipping of 8 bases.

**Conclusions:**

Here we report on three unrelated families presenting with FADS caused by four *TTN* variants. In addition, our study demonstrates that pathogenic meta transcript-only *TTN* variant can lead to defects which is recognizable prenatally in a recessive manner.

**Supplementary Information:**

The online version contains supplementary material available at 10.1186/s12920-024-01946-z.

## Introduction

Congenital myopathy is a group of diseases with heterogeneous etiology and a wide spectrum of phenotypes [1]. The widespread use of ultrasound screening leads to a high frequency of diagnosis of fetal muscle abnormalities. A diagnosis of severe myopathy always invites considerations regarding termination of pregnancy and inevitably raises concerns about the risk of recurrence in the future pregnancies. Identification of the potential genetic causes facilitates accurate genetic counseling as well as alleviating concerns. The application of next-generation sequencing (NGS) techniques has enabled the genetic basis of neuromuscular diseases to be elucidated, allowing more disease-causing variants to be characterized.

The *TTN* gene (OMIM: 188,840) encodes titin, a giant sarcomeric protein that plays an important functional and structural role in the sarcomere [2–5]. Pathogenic variants in *TTN* were reported to cause a range of skeletal muscle and cardiac disorders, such as dilated cardiomyopathy-1G (CMD1G; OMIM: 604,145), familial hypertrophic cardiomyopathy-9 (CMH9; OMIM: 613,765), tibial muscular dystrophy (TMD; OMIM: 600,334), myofibrillar myopathy-9 with early respiratory failure (MFM9; OMIM: 603,689), and limb-girdle muscular dystrophy type 2 J (LGMD2J; OMIM: 608,807) [6–10]. As one of the most complex human genes, *TTN* contains 364 exons and four major structural regions (Z disk, I band, A band, and M band), giving rise to a large number of alternatively spliced transcripts [11, 12]. The inferred complete (IC) transcript (NM_001267550.2), also known as meta-transcript, is a theoretical isoform that includes all putative exons (except the exon 48 in transcript NM_133379.5) [13–15]. The exons not included in any of the recognized postnatal skeletal muscle isoform (N2A) and cardiac muscle isoforms (N2B, N2BA, Novex-1, Novex-2, and Novex-3) are defined as meta transcript-only exons and are generally thought to be expressed primarily during embryonic development, although some of them have low expression in the postnatal setting [1, 7, 13, 15]. Some studies have proposed that the onset and severity of *TTN*-related diseases are related to the type and location of the variants [1, 16].

Herein, we report on the investigation of three unrelated families with fetal akinesia deformation sequence (FADS). Three novel *TTN* variants with a common meta transcript-only variant *in trans* were identified in our study.

## Clinical reports

### Family 1

**Fig. 1:**
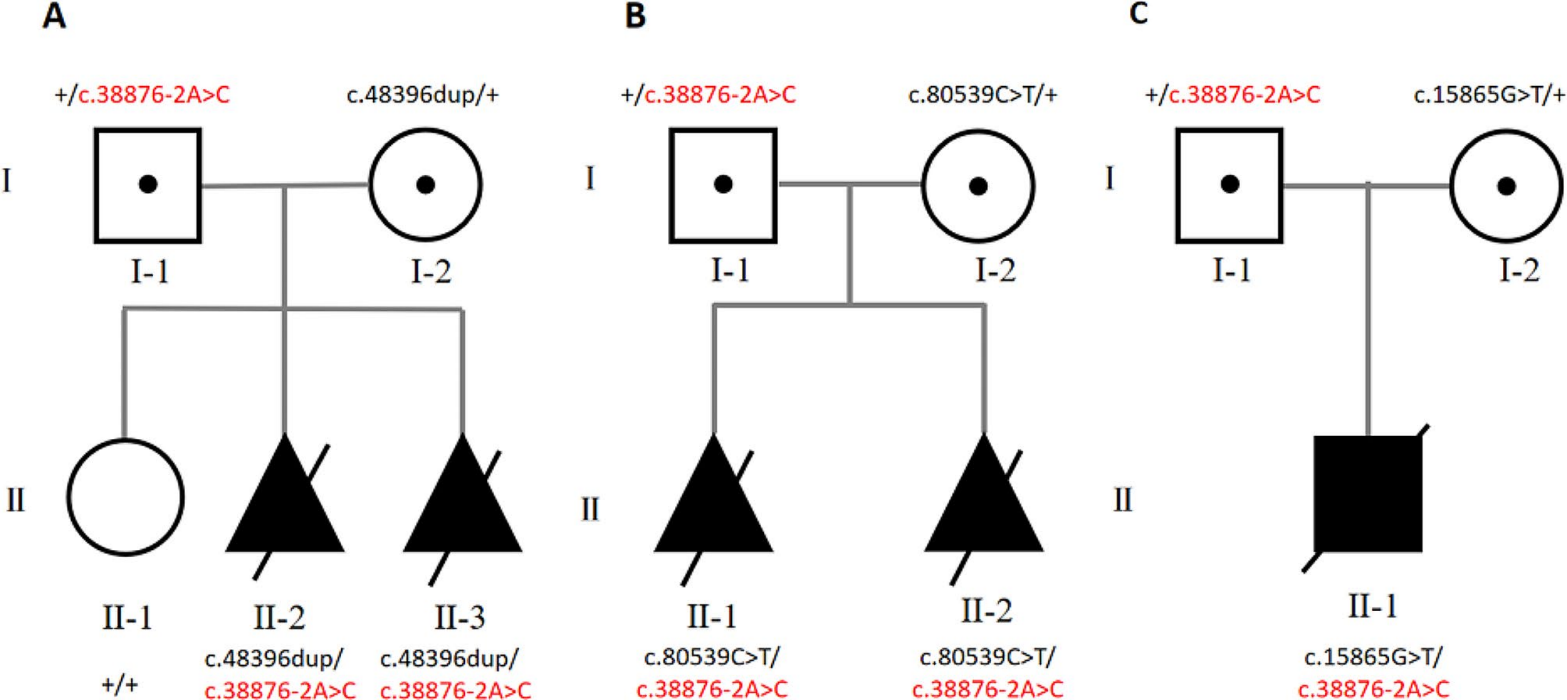
Pedigree of the three families and segregation of the recessive *TTN* variants, including the common c.38,876–2 A > C haplotype. (**A**) Family 1; (**B**) Family 2; **C** Family 3

The healthy and non-consanguineous couple has no family histories of neuromuscular diseases or cardiomyopathies. Their first daughter was healthy (Fig. 1A, II-1). During the second pregnancy (Fig. 1A, II-2), the women reported poor fetal movement. Fetal ultrasound in the 22nd week of gestation showed a thickened nuchal fold of 10 mm (normally < 6 mm in the second trimester) and sustained flexed elbows with no joint movements. The pregnancy was terminated in the 24th week after the couple received genetic counsel. Chromosomal karyotype and chromosomal microarray analysis (CMA) on amniotic fluid showed no significant findings.

**Fig. 2:**
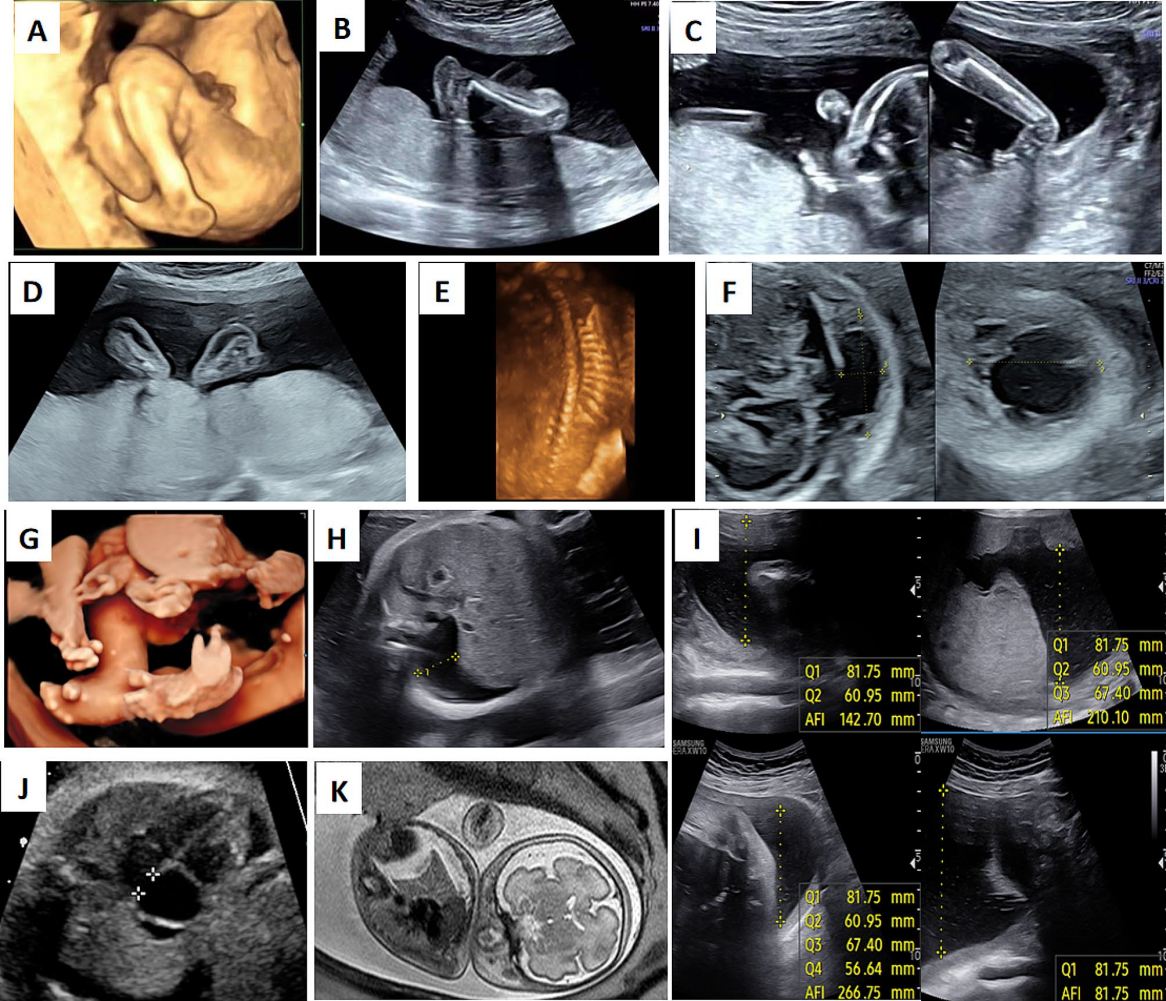
Imaging findings. (**A-C**) fetal ultrasound images from F1-II0.3. (**A**) ankle and knee contractures; (**B**) the left foot presented with rocker bottom deformity; (**C**) wrist contractures. (**D-F**) Fetal ultrasound images from F2-II0.2. (**D**) the abnormal fetal posture with the palms of the feet facing each other; (**E**) the abnormal curvature of the fetal spine; (**F**) thickened nuchal fold with cystic the dark area inside. (**G-K**) Imaging findings from F3-II0.1. (**G**) flexed position of all limb joints; (**H**) a right pleural effusion with a width of about 16 mm; (**I**) mild pericardial effusion with a width of 3.6 mm; (**J**) widened extracerebral space and mild deformation of the cerebral parenchyma showed by MRI; (**K**) polyhydramnios with an amniotic fluid index (AFI) of 266 mm and a maximum depth of 95 mm

Unfortunately, similar fetal abnormalities were observed during their third pregnancy (Fig. 1A, II-3). Ultrasound screening in the 22nd week of pregnancy revealed abnormal fetal posture with persistent limb joint contractures, rocker-bottom feet, and significantly decreased fetal movement (Fig. 2A-C). The couple terminated the pregnancy in the 23rd week of gestation after genetic counseling.

### Family 2

The couple was healthy and non-consanguineous, and their detailed family history was noncontributory. During their first pregnancy (Fig. 1B, II-1), fetal ultrasound in the 25th week of gestation revealed polyhydramnios, hydrops fetalis, scoliosis, flexion of the limbs, and lack of movement. The pregnancy was terminated after genetic counseling. CMA performed on the fetal tissue revealed no pathogenic copy number variants (CNVs). One year later, during the second pregnancy (Fig. 1B, II-2), the tragedy repeated: hydrops fetalis, cystic hygroma, scoliosis, flexion of the limbs, and poor fetal movement were revealed by ultrasonography (US) performed in the 24th week of gestation (Fig. 2D-F). Again, the couple terminated the pregnancy.

### Family 3

It was a healthy, non-consanguineous couple, with the age of 28 for the husband and 27 for the wife. This was their first pregnancy (Fig. 1C, II-1). Fetal ultrasound in the 23rd week of gestation indicated contractures of all four limbs, right-sided pleural effusion, and polyhydramnios. All of the fetal abnormalities were confirmed by the following ultrasound in the 31st week (Fig. 2G-I). Pericardial effusion (PEFF) and widened extracerebral space were revealed subsequently by fetal echocardiogram and magnetic resonance imaging (MRI), respectively (Fig. 2J-K). The pregnancy was terminated upon parental request. CMA performed on the fetal tissue didn’t find any pathogenic CNVs.

## Methods

### WES and data analyses

Genomic DNA was extracted from the muscle tissue of the five aborted fetuses and peripheral blood from the couples. Proband-WES was performed in family C while trio-WES was selected for the other two families. The database of single nucleotide polymorphisms (dbSNP, http://www.ncbi.nlm.nih.gov/snp), the 1000 Genomes Project database (http://browser.1000genomes.org), and genome Aggregation Database (gnomAD v4.0.0, http://gnomad.broadinstitute.org/) was used for searching the minor allele frequencies (MAF < 0.1%) of all known variants. Online bioinformatics tools Mutation Taster (http://www.mutationtaster.org), Polyphen-2 (http://genetics.bwh.harvard.edu/pph2), SIFT (http://sift.jcvi.org), REVEL (https://sites.google.com/site/revelgenomics/), CADD(https://cadd.gs.washington.edu), and SpliceAI (https://github.com/lllumina/SpliceAl) were used to predict the effects of the variants. The pathogenicity of the variants was determined according to the American College of Medical Genetics and Genomics (ACMG) guidelines [17]. Sanger sequencing was performed on family members to confirm segregation and carrier status.

### RNA isolation and expression analysis via reverse transcription and PCR

We extracted mRNA from 10 ml of whole blood from the father of family 1 following the manufacturer’s manual (RNAiso Plus, TaKaRa, Japan). Nano Drop 2000 (Thermo Scientific) was used to determine the RNA concentration. The extracted mRNA was synthesized into cDNA (PrimeScriptTM RT reagent Kit, TaKaRa, Japan). PCR was performed using the following primers: 5’-AAAGCCAGAAGCTCCACCTC-3’ (forward primer at the start of exon 192) and 5’-CTCAGGCTCCTCGAACACTT-3’ (reverse primer at the end of exon 205). The cDNA PCR product was visualized by 2% agarose gel electrophoresis and analyzed by Sanger sequencing on the ABI3730xl Genetic Analyzer (Applied Biosystems).

## Results

### Mutation detection

**Fig. 3:**
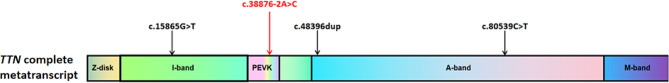
A schematic representation of main titin regions encoded by the inferred complete meta-transcript and the corresponding location of variants identified in the current study

Heterozygous variant c.38,876–2 A > C was found in all the affected fetuses (Fig. 1). The variant c.38,876–2 A > C, a meta transcript-only variant, is a canonical splicing variant and lies within the proline-glutamine-valine-lysine (PEVK) repeat region (Fig. 3). It was presented in the gnomAD database at a very low frequency of 0.000005611 (9/1,604,030 alleles) in total of the population.

Three truncating variants: c.48396dup (p. Asn16133Ter), c.80,539 C > T (p. Gln26847Ter), and c.15865G > T (p. Glu5289Ter) were found to co-segregate with the c.38,876–2 A > C in the three families, respectively (Fig. 1). Variants c.48396dup (p. Asn16133Ter) and c.80,539 C > T (p. Gln26847Ter) are located in the A-band, while c.15865G > T (p. Glu5289Ter) lies within the I-band (Fig. 3). All three variants are predicted to induce loss of function (LoF) for the encoded titin protein. The frequency of c.48396dup and c.80,539 C > T in the gnomAD v4.0 was 0.000001594 (1/627,338 alleles) and 0.000006580 (1/151,974 alleles), respectively. And c.15865G > T was not recorded in the gnomAD v4.0. According to the ACMG guidelines, c.38,876–2 A > C was classified as likely pathogenic (PM2_Supporting + PVS1_Moderate + PM3_Strong + PP1), c.48396dup was classified as pathogenic (PVS1 + PM2_Supporting + PP1), c.80,539 C > T and c.15865G > T were classified as likely pathogenic (PVS1 + PM2_Supporting). The *TTN* variants are reported and numbered with exons using the inferred meta-transcript as the reference (NM_001267550.2).

The clinical and molecular features of the three families are summarised in Supplemental Table 1.

### RT-PCR and sequencing analysis

To confirm the effect of c.38,876–2 A > C on *TTN* mRNA, the cDNA from the carrier sample was amplified using primers toward the region between exon 192 and exon 205.

**Fig. 4:**
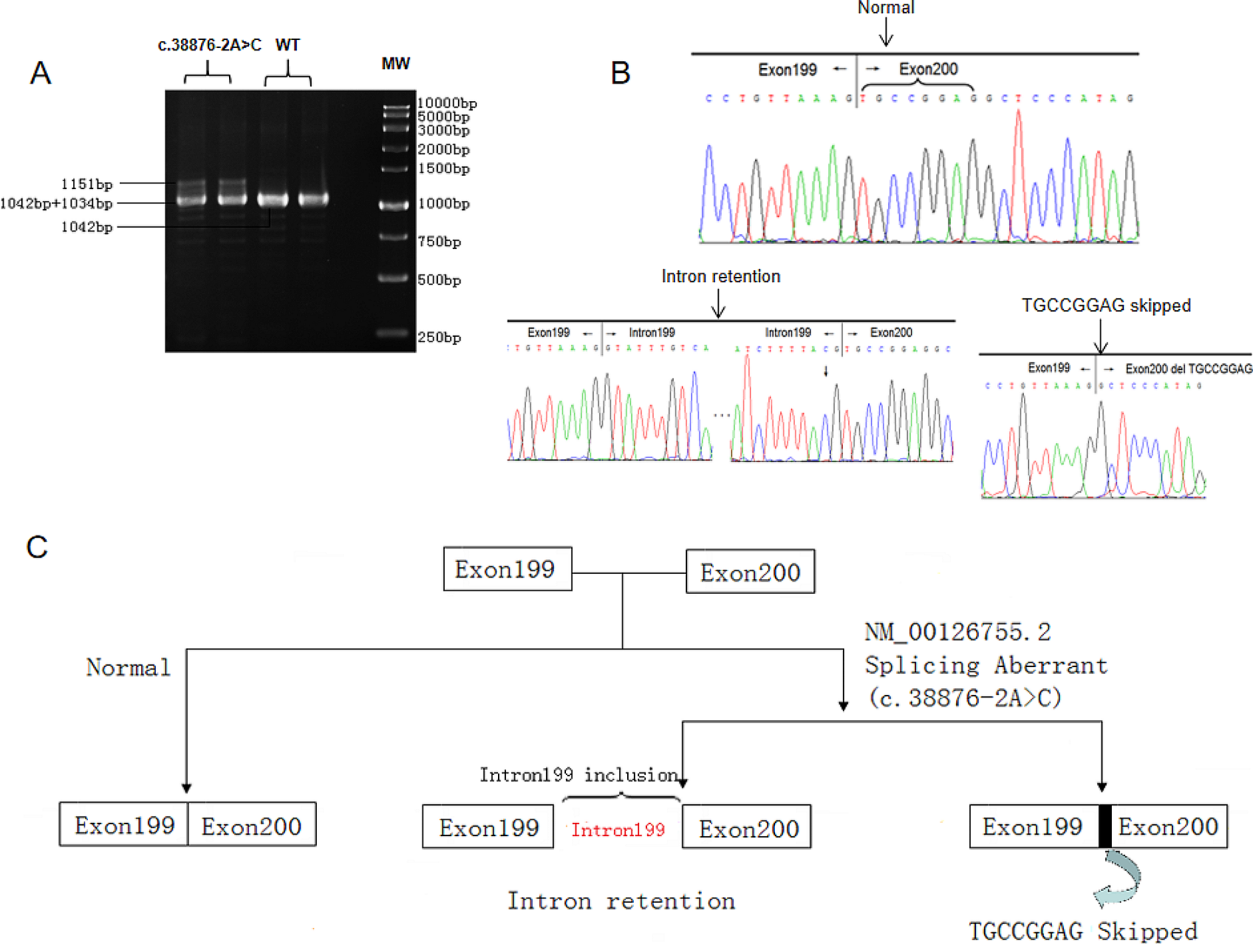
Transcript analyses of *TTN* variant c.38,876–2 A > C. (**A**) Display of cropped gels for the RT-PCR fragments, on the right molecular weight (MW) marker. A band of 1042 bp corresponding to the wild-type (WT), a band of 1151 bp corresponding to a retention of intron 199, and a band of 1034 bp corresponding to the skipping of 8 nucleotides. (**B**) Sanger sequencing of the splicing variant resulting from the retention of the whole intron 199 and the skipping of 8 nucleotides of Exon 200. (**C**) Schematic representation of the abnormal splicing combining the results of RT-PCR and Sanger sequencing

Agarose gel electrophoresis showed that the control sample had only one band with the length of 1042 bp, while the carrier sample showed three bands with lengths of 1034 bp, 1042 bp, and 1151 bp, respectively (Fig. 4a). Sanger sequencing for the abnormal cDNA revealed skipping of 8 nucleotides and retention of intron 199 (Fig. 4b and c). The full length original blot can be seen in Supplemental Fig. 1.

## Discussion

Titin is the largest sarcomeric protein and is organized into four structurally and functionally distinct regions, including Z-disk, I-band, A-band, and M-disk (Fig. 3), each of which has a largely independent functional role [18]. The I-band region is mainly composed of repetitive immunoglobulin (Ig) domains and the PEVK region, which is rich in proline (P), glutamic acid (E), valine (V), and lysine (K), and unravels when stretched to give the muscle elasticity [11, 19]. Exons within the PEVK region are extensively alternatively spliced, regulating passive tension and muscle elasticity.

Previous studies have shown the genotype-phenotype correlations: hereditary myopathy with early respiratory failure (HMERF) is specifically caused by missense variants in the exon 344; tibial muscular dystrophy (TMD) is caused by heterozygous variants in the last exon [20, 21]; all remaining *TTN*-related skeletal muscle diseases are recessively inherited and present a phenotypic spectrum of severe early-onset disorders collectively termed “congenital titinopathies”, which includes core myopathy with heart disease, centronuclear myopathy, early onset myopathy with fatal cardiomyopathy, and arthrogryposis multiplex congenita [3, 4, 20, 22]. There are several reports of congenital myopathies associated with variants within meta transcript-only exons [1, 3, 7, 13, 14, 23, 24], while variants within meta transcript-only introns is rarely reported [2, 16]. Previous studies showed that the meta transcript-only exons are observably more highly expressed in fetal muscle than adult muscle [2, 15]. It is hypothesized that meta transcript-only variants specifically and selectively affect fetal isoforms, disrupting the development and assembly of fetal muscle, leading to prenatal or congenital severe myopathic phenotypes [3, 15, 16]. In our study, five fetuses with a common meta transcript-only canonical splice site variant all presented with arthrogryposis and a lack of movement, providing a good validation of the above hypothesis.

The c.38,876–2 A > C variant has been previously reported as an incidental finding [25]. And it is not thought to contribute to the fetal phenotypes including skin oedema, pleural effusion, and talipes equinovarus. However, skin oedema and pleural effusion were all observed in our study. We think these are not incidental findings but part of the phenotype. Meanwhile it suggests that characterization of a complete spectrum of *TTN*-related diseases remains difficult and needs more studies to provide further evidence. Variations in splicing receptor and donor sites are usually considered to lead to abnormal splicing [17]. RNA analysis should be performed since it is a powerful tool to verify the result. It is a great pity that we missed the opportunity to collect muscle tissue from the fetuses. As a remedy, we took blood sample from the carrier for RNA analysis. Despite very low levels of expression in peripheral blood, we succeeded in obtaining the cDNA sequences and confirmed two types of abnormal splicing caused by the mutation. Both abnormal splicing outcomes lead to a frameshift predicted to trigger nonsense mediated decay and result in loss of function (LoF) for the *TTN* transcript. Since all outcomes are LoF, PVS1 can be applied instead of PVS1_moderate, according to the relevant guidelines [26], and the variant c.38,876–2 A > C is reclassified as pathogenic.

Previous data suggested that variants in exons not expressed at significant levels in cardiac tissue are not associated with cardiomyopathy. Patients with two pathogenic variants predicted to affect both of the cardiac isoforms (N2BA and N2B) have a significantly higher risk of cardiac involvement than those with other combinations of *TTN* variants [3, 13, 27]. To date, no cardiac phenotype has been reported in individuals with meta transcript-only pathogenic variants [1, 2, 13, 23, 24]. Here, according to a detailed prenatal ultrasound, none of the five fetuses showed signs of cardiac structural malformation.

Heterozygous titin-truncating variants (TTNtv) in exons that are constitutively expressed in cardiac tissue have been identified as the most common genetic cause of dominant or sporadic dilated cardiomyopathy (DCM) [19, 28–32]. As the variants c.48396dup and c.80,539 C > T affect both N2B and N2BA, the two heterozygous carriers (Family 1: I-2 and Family 2: I-2) were referred for cardiac surveillance and the echocardiogram showed normal structural with no sign of cardiomyopathy. The genotype-phenotype correlation for cardiac involvement remains unclear. However, age should not be excluded, as TTNtv-induced cardiomyopathy shows an age-related penetrance [13, 16, 33].

The five fetuses exhibited the major FADS phenotypes such as reduced fetal movements, polyhydramnios, hydrops fetalis, limb contractures, scoliosis, and pleural effusion. One fetus also showed widened extracerebral space which has been reported in very few cases [13], but whether it is caused by *TTN* mutation is unclear. Although the widespread use of NGS has enabled the discovery of an increasing number of variants in *TTN*, characterization of a complete spectrum of *TTN*-related diseases remains difficult due to the high frequency of *TTN* variants, incomplete penetrance, and as yet unidentified underlying disease mechanisms.

## Conclusions

Our finding not only clarify the etiology of the fetal anomaly in the three families, but also help guide their next pregnancy. Pre-implantation genetic diagnosis is a good choice for them. Significant polyhydramnios, arthrogryposis, and reduced fetal movements often indicate neuromuscular disease, and we recommend autosomal recessive congenital titinopathy as one of the differential diagnoses.

### Electronic supplementary material

Below is the link to the electronic supplementary material.

Supplementary Material 1

Supplementary Material 2

## Data Availability

The raw sequencing data is available in the Sequence Read Archive (SRA) submission: PRJNA1057424.
