# Supplementary material for: Identification of four TTN variants in three families with fetal akinesia deformation sequence
Source: BMC Med Genomics. 2024 Jun 27;17:170. doi: 10.1186/s12920-024-01946-z (PMC11212154; doi:10.1186/s12920-024-01946-z)
Supplement: Supplementary file 1 — Supplementary Material 1 [file 12920_2024_1946_MOESM1_ESM.docx]

|  | **Reduced foetal movements** | **Polyhydramnios** | **Hydrops fetalis** | **Limb contractures** | **Scoliosis** | **Pleural effusion** | **Thickened nuchal fold** | **Widened**  **extracerebral spaces** | **Pericardial effusion** | **Allele 1** | | | **Allele 2** | | |
| --- | --- | --- | --- | --- | --- | --- | --- | --- | --- | --- | --- | --- | --- | --- | --- |
|  |  |  |  |  |  |  |  |  |  | **Variant description** | **Exon/Intron--Region** | **Isoforms** | **Variant description** | **Exon/Intron**  **--Region** | **Isoforms** |
| **Family 1** | ＋ | － | － | ＋ | － | － | ＋ | － | － | c.38876-2A>C/- | Int199--  PEVK | Meta transcript-only | c.48396dup: p.Asn16133ter | 258--  A-Band | N2A, N2B, and N2BA |
| **Family 2** | ＋ | ＋ | ＋ | ＋ | ＋ | ＋ | － | － | － | c.38876-2A>C/- | Int199--  PEVK | Meta transcript-only | c.80539C>T:  p.Gln26847Ter | 326--  A-Band | N2B and N2BA |
| **Family 3** | ＋ | ＋ | － | ＋ | － | ＋ | － | ＋ | ＋ | c.38876-2A>C/- | Int199--  PEVK | Meta transcript-only | c.15865G>T:  p.Glu5289Ter | 54--  I-Band | N2A and N2BA |
