# Supplementary figures and images for: Identification of four TTN variants in three families with fetal akinesia deformation sequence
Source: BMC Med Genomics. 2024 Jun 27;17:170. doi: 10.1186/s12920-024-01946-z (PMC11212154; doi:10.1186/s12920-024-01946-z)

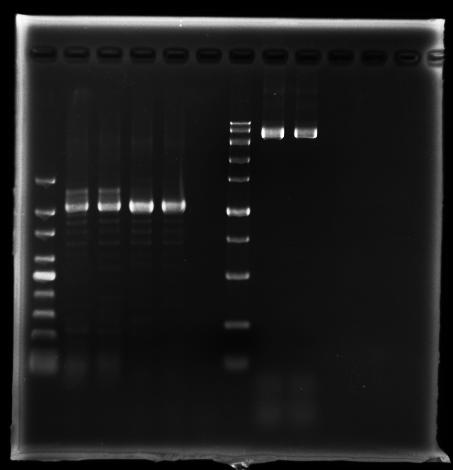

Supplement: Supplementary file 2 — Supplementary Material 2 [file 12920_2024_1946_MOESM2_ESM.png]
